# Supplementary material for: Diagnostic Performance of a Fecal Immunochemical Test-Based Colorectal Cancer Screening Program According to Ambient Temperature and Humidity
Source: Cancers (Basel). 2022 Feb 23;14(5):1153. doi: 10.3390/cancers14051153 (PMC8909312; doi:10.3390/cancers14051153)
Supplement: Supplementary file 1 [file cancers-14-01153-s001.zip › cancers-1544367-supplementary.pdf]

Diagnostic Performance of a Fecal Immunochemical Test-Based Colorectal Cancer Screening Program According to Ambient Temperature and Humidity

Gemma Ibáñez-Sanz; Núria Milà; Núria Vives; Carmen Vidal; Gemma Binefa; Judith Rocamora; Carmen Atencia; Víctor Moreno; Rebeca Sanz-Pamplona; Montse Garcia and on behalf of the MSIC-SC research group

Table S1. Temperature and storage time of the faecal immunochemical tests

Stage	Temperature	Controlled temperature	Storage time[1]
Sample collection	2-8°C[2]	No	Maximum 3 days
Delivery to pharmacy	Ambient temperature	No	
Pharmacy	2-8°C[3]	Yes	Maximum 2 days
Transport to dispenser warehouse	2-8°C[4]	Yes	
Dispenser warehouse	2-8°C[4]	Yes	Maximum 3 days
Transport to laboratory	2-8°C[4]	Yes	Maximum 1 day
Laboratory	2-8°C[4]	Yes	Maximum 3 days

[1]Maximum time according to protocol; [2]In case of not immediate return; [3]In compliance with Decree 168/1990, 3 July, which establishes the technical-sanitary requirements that must be met; [4]In compliance with the corresponding good manufacturing practices as well as Royal Decree 782/2013 11 October on the distribution of medicines for human use.

Table S2a. Baseline characteristics of participants according to temperature

	Maximum ambient temperature	
	**≤24 ºC** *N=63964*	**>24ºC** *N=28309*
Sex		
Female	35130 (54.9%)	15844 (56.0%)
Male	28834 (45.1%)	12465 (44.0%)
Age (years)		
50-59	31497 (49.2%)	13083 (46.2%)
60-69	32467 (50.8%)	15226 (53.8%)
Screening episode		
Initial	38049 (59.5%)	15811 (55.9%)
Successive	25915 (40.5%)	12498 (44.1%)
Socioeconomic Score		
0-39 (least deprived)	6054 (9.5%)	995 (3.5%)
39-51	31919 (49.9%)	3659 (12.9%)
52-100 (most deprived)	25991 (40.6%)	23655 (83.6%)

Table S2b. Baseline characteristics of participants according to relative humidity

	Maximum ambient humidity	
	**≤89%** *N=81379*	**>89%** *N=10894*
Sex		
Female	44986 (55.3%)	5988 (55.0%)
Male	36393 (44.7%)	4906 (45.0%)
Age (years)		
50-59	38978 (47.9%)	5602 (51.4%)
60-69	42401 (52.1%)	5292 (48.6%)
Screening episode		
Initial	48495 (59.6%)	5365 (49.2%)
Successive	32884 (40.4%)	5529 (50.8%)
Socioeconomic Score		
0-39 (least deprived)	5610 (6.9%)	1439 (13.2%)
39-51	28262 (34.7%)	7316 (67.2%)
52-100 (most deprived)	47507 (58.4%)	2139 (19.6%)

Table S3. Logistic regression of the probability of positive screening tests by quarter year (95% CI)

	**Number of FITs** *N=5048*	**OR[2]**	**95% CI**	**P-value**
Sex				
Female	2185 (43.3%)	1.00		<0.001
Male	2863 (56.7%)	1.67	1.58-1.77	
Age[1] (years)	60.7 (5.8)	1.03	1.03-1.04	<0.001
Screening episode				
Initial	3306 (65.5%)	1.00		<0.001
Successive	1742 (34.5%)	0.68	0.64-0.72	
Socioeconomic score[1] (mean (SD))	53.5 (10.9)	1.01	1.00-1.01	<0.001
Quarter year of FIT performance				
1st quarter (January-March)	1675 (33.2%)	1.00		
2nd quarter (April-June)	1007 (19.9%)	0.91	0.84-0.98	0.02
3rd quarter (July-September)	449 (8.9%)	0.85	0.76-0.96	0.004
4th quarter (October-December)	1917 (38.0%)	0.90	0.84-0.97	0.003

[1]Continuous variable; [2]All variables shown are included in the multivariate analysis. FIT: faecal immunochemical test.

Table S4. Logistic regression of the probability of having an advanced neoplasia diagnosed (Screen-detected CRCs, high-risk lesions and intermediate-risk lesions) by quarter year

	**No advanced neoplasia** ***N=2367***	**Advanced neoplasia** ***N=2151***	**OR[2]**	**95% CI**	**P-value**
Sex					
Female	1278 (54.0%)	678 (31.5%)	1.00		
Male	1089 (46.0%)	1473 (68.5%)	2.53	2.23-2.85	<0.001
Age[1] (mean (SD)) (years)	60.6 (5.8)	60.8 (5.8)	1.01	1.00-1.02	0.01
Screening episode					
Initial	1422 (60.1%)	1515 (70.4%)	1.00		
Successive	945 (39.9%)	636 (29.6%)	0.63	0.56-0.72	<0.001
Socioeconomic score[1] (mean (SD))	53.3 (10.8)	53.5 (11.1)	1.00	1.00-1.01	0.38
Quarter year of FIT performance					
1st quarter (January-March)	797 (33.7%)	688 (32.0%)	1.00		
2nd quarter (April-June)	439 (18.5%)	446 (20.7%)	1.17	0.98-1.38	0.08
3rd quarter (July-September)	203 (8.6%)	191 (8.9%)	1.07	0.84-1.35	0.59
4th quarter (October-December)	928 (39.2%)	826 (38.4%)	1.02	0.89-1.18	0.76

[1]Continuous variable; [2]All variables shown are included in the multivariate analysis. FIT: faecal immunochemical test
